# Kourami: graph-guided assembly for novel human leukocyte antigen allele discovery

**DOI:** 10.1186/s13059-018-1388-2

**Published:** 2018-02-07

**Authors:** Heewook Lee, Carl Kingsford

**Affiliations:** Computational Biology Department, School of Computer Science, Carnegie Mellon University, Pittsburgh, PA USA

## Abstract

Accurate typing of human leukocyte antigen (HLA) is important because HLA genes play important roles in immune responses and disease genesis. Previously available computational methods are database-matching approaches and their outputs are inherently limited by the completeness of already known types, making them unsuitable for discovery of novel alleles. We have developed a graph-guided assembly technique for classical HLA genes, which can construct allele sequences given high-coverage whole-genome sequencing data. Our method delivers highly accurate HLA typing, comparable to the current state-of-the-art methods. Using various data, we also demonstrate that our method can type novel alleles.

**Electronic supplementary material:**

The online version of this article (10.1186/s13059-018-1388-2) contains supplementary material, which is available to authorized users.

## Background

Human leukocyte antigen (HLA) genes are crucial in the regulation of the immune system as they encode for the major histocompatibility complex (MHC) consisting of cell surface proteins that control the adaptive immune response. HLA genes are also known to play important roles in transplant rejection as well as infectious and autoimmune diseases [1–4]. For these reasons, accurate HLA typing is important both in clinical and research settings. HLA typing is considered challenging because of the hyper-polymorphic nature of the HLA region in the human genome. Such high polymorphism in the HLA region is thought to be maintained by strong balancing selection promoting genetic diversity [5, 6]. Especially with personal genome sequencing becoming widely common, better computational methods are needed to provide rapid and inexpensive typing with high accuracy.

Traditionally, HLA typing or categorization was done by laborious serology-based methods that screen for HLA antibodies in a donor/receiver pair. With the birth of DNA sequencing and the polymerase chain reaction (PCR), molecular typing assays, such as specific oligonucleotide probe hybridization, sequence-specific primer amplification, and sequence-based typing (SBT), have been developed [7]. The SBT method can be used with either Sanger sequencing or next-generation sequencing (NGS) techniques. By using specific primers for target enrichment prior to sequencing, SBT delivers accurate and reliable typing of HLA alleles. However, all of the above molecular typing assays require a specially designed set of probes or primers.

**Fig. 1:**
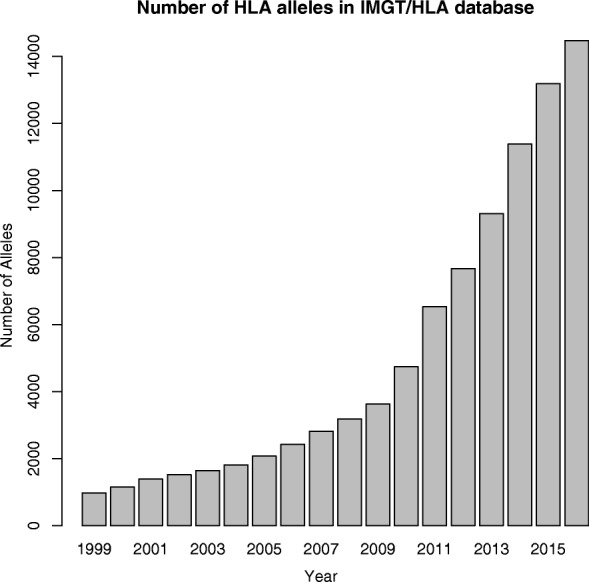
The number of alleles in the IPD-IMGT/HLA database by year from 1999 to 2016. The database releases updates four times a year (January, April, July, and October) and the plot is based on the number of alleles from all the April releases reported on the statistics page of the IPD-IMGT/HLA website (http: //www.ebi.ac.uk/ipd/imgt/hla/stats.html). HLA human leukocyte antigen

With the increasing availability of personal whole-genome sequencing (WGS) services, the availability of accurate computational HLA typing methods that do not require additional experiments can be valuable. Challenges in computational HLA typing are mainly driven by the high level of polymorphism found in the HLA region in the human genome. Over 30 genes are maintained in the IPD-IMGT/HLA database [8] and six to eight classical HLA genes (HLA-A, -B, -C, -DQA, -DQB, and -DRB) are routinely used for HLA typing in clinical settings. More than 15,000 known alleles (just for these classical genes) have been reported in the database and the number of alleles is growing rapidly (Fig. 1). Also, the known alleles share high sequence similarities, where many alleles differ just by a base-pair substitution. Thus, it is challenging to pinpoint correctly an individual’s HLA types among the known alleles using WGS data [9].

Previously developed enrichment-free computational methods can use WGS, whole-exome sequencing (WES), or transcriptome sequencing (RNA-seq) without the use of HLA-enriched data, unlike SBT. However, many do not provide typing accuracy comparable to what SBT provides [10], with the exception of a recently developed method, HLA*PRG [11]. However, the high accuracy of HLA*PRG comes at the cost of speed.

These computational methods either use one or both of two major techniques (alignment and assembly) to compare reads accurately when correcting HLA genes and inferring allele types. For computational HLA typing of NGS data, an alignment-based method was first developed for HLA typing using targeted sequencing of class I genes [12]. This method incorporated the Bayesian genotyping information from a widely used toolkit GATK [13] and additionally modeled the phasing by considering pairs of adjacent variant sites. Other computational typing methods that can work with enrichment-free data were developed later. Alignment-based methods, such as seq2HLA [14], HLAforest [15], and PHLAT [16], first attempt to assign NGS reads correctly to HLA loci using various stringent quality filtering procedures to selectively discard erroneous reads with low alignment scores, then use probabilistic variant-calling approaches similar to that in [12] to infer the closest matching allele in the database. Unlike most alignment-based methods, HLA-VBSeq [17] uses variational Bayesian inference to assign reads correctly to alleles based on alignment, as done in solutions to the RNA-seq quantification problem [18]. OptiType [19] also uses alignment; however, it formulates the typing problem as an optimization problem via integer linear programming (ILP), where the objective is to find a solution set of alleles such that the number of mapped reads is maximized with constraints that each locus requires at least one allele (homozygous) and at most two alleles (heterozygous). The recently published xHLA [20] also adopts OptiType’s ILP formulation but uses protein-level alignment of the typing exons as it focuses on four-digit resolution typing (protein-level). After solving the optimization problem restricted to just the typing exons, it uses iterative refinement steps to select alleles that further maximize the number of mapped reads when considering all exons. Assembly-based methods, such as HLAminer [21] and HLAreporter [22], first construct longer-than-read contigs using de novo assembly techniques and search for best matches among known alleles. Direct assembly of haplotypes by traditionally available de novo or reference-based assemblers is severely confounded by the high level of polymorphisms. Often, assembly-based methods combine the techniques used in alignment-based methods. For example, HLAreporter first uses read alignment to known alleles and identifies reads that are likely from HLA regions prior to its assembly step. Most recently, HLA*PRG [11] improved the accuracy of alignment-based methods by representing the database alleles as graphs and using a more sensitive alignment on the graphs. It first aligns extracted reads likely from the HLA region to population reference graphs [23] that encode all known alleles and it outputs the most likely alleles from the database.

One common aspect of the enrichment-free computational HLA typing methods is that they are all primarily driven by the *finding-the-nearest-match* paradigm. Their goal is to find the best-matching alleles to HLA genes of a test individual in a preexisting database of known entries. Given the sequencing data of an individual, such a typing scheme outputs the best-matching alleles for each HLA gene. This typing strategy is limited by the completeness of the collection of known alleles, as it cannot detect novel alleles missing in the database of known types. We collectively refer to these approaches as *database-matching* methods. Novel alleles can possibly have protein-coding changes that may have a profound impact for organ transplantation and disease association. One might argue that there are already many known alleles and that the chance of finding novel alleles is low. However, the number of known alleles in the IPD-IMGT/HLA database is still increasing rapidly (Fig. 1). There is continuing effort among immunogenetics communities to study rare and novel alleles. For example, the *International HLA and Immunogenetics Workshop* has been organizing projects to investigate and collect rare and novel alleles since the 15th workshop in 2002. Immunogenetics-related journals, such as the *International Journal of Immunogenetics* and *HLA* (formerly known as *Tissue Antigens*), have a dedicated section where new alleles are announced in every issue.

For these reasons, it is important to be able to recover HLA sequences at 1-bp resolution to enable novel allele discovery as done in SBT. To achieve this goal, we present a graph-guided assembly technique called Kourami that constructs full sequences for the peptide-binding domain (exons 2 and 3 for class I and exon 2 for class II HLA genes, which are regions typed by the SBT methods) using a modified partial-order graph (POG) [24] as a guide. The graph representation compactly captures variant regions among related sequences to take advantage of known alleles, and it also provides a framework in which to incorporate information from the sequencing reads to encode novel alleles.

In fact, graphs have long been used to represent varying regions. For example, de Bruijn graphs [25] and string graphs [26] have been extensively used in fragment assembly. More recently, graph-based representations of populations of genomes, which are also related to POG, have been an active research area [23, 27–30]. Our method is the first that directly assembles both haplotypes of HLA genes rather than inferring the best-matching alleles in the database. For known alleles, we show that Kourami can correctly type with high accuracy (>98%), equaling that of the state-of-the-art database-matching method, across various WGS data sets, such as simulated data, Illumina Platinum Genomes, and high coverage WGS from the 1000 Genomes Project. At the same time, Kourami takes only a fraction of the time compared to other available methods with a moderate use of memory.

Kourami is the first HLA typer to be able to assemble novel alleles that do not appear in the database. It does this by treating the HLA typing problem as an instance of graph-guided assembly, where the known alleles are combined into a graph that is used to guide the assembly of new alleles. Kourami, therefore, also represents an early example of how a population of reference sequences can be used during genome assembly. We systematically show that Kourami is very accurate in constructing novel alleles by performing leave-one-out experiments where a known allele is artificially removed from the allele database. Kourami is able to reconstruct 98% of these alleles perfectly.

## Results

### HLA typing nomenclature

The current HLA allele nomenclature [31] uses a hierarchical numbering system with four major levels of hierarchies. From the highest to the lowest category, it annotates allele groups (two-digit resolution), protein sequence (four-digit resolution), exon sequence (six-digit resolution), and intron sequence (eight-digit resolution). For example, if two alleles encode an identical protein, they will have the same numbers for the first two levels of (four-digit) hierarchies. In practice, HLA typing is often carried out at either the protein or exon level. Furthermore, the current gold standard, SBT, types just the exons that are responsible for encoding the peptide-binding domain (exons 2 and 3 for class I genes and exon 2 for class II genes). Using only the subset of exons creates ambiguous alleles where two or more alleles share an identical sequence over these exons but differ in other regions. These ambiguous sequences are grouped as a six-digit G allele. Similarly, four-digit P grouping is used for the alleles that share the same amino acid sequence over these exons. Our method provides a fully assembled sequence covering these exons and also outputs a six-digit G resolution typing result by selecting known alleles that have the smallest edit distance to the assembled sequences. Like many other HLA tools, we focus on the routinely typed classical genes (HLA-A, -B, -C, -DQA1, -DQB1, and -DRB1).

### Overview of method

**Fig. 2:**
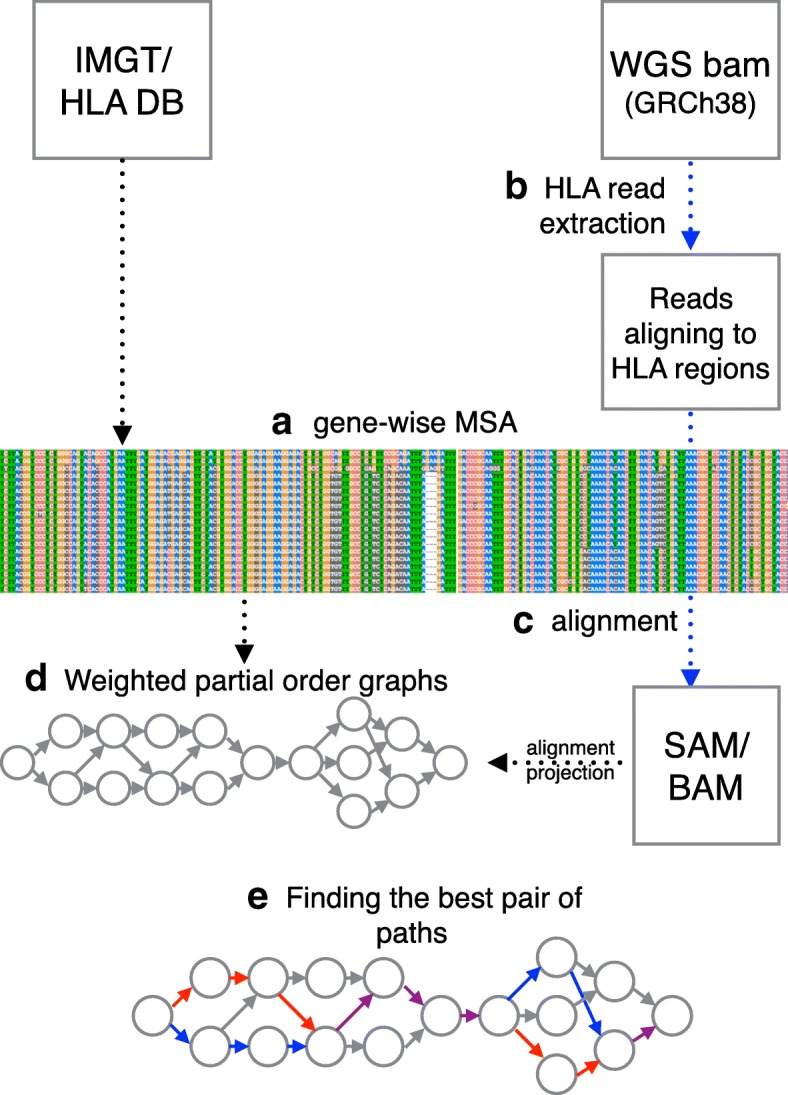
Overview of our method. **a** A gene-wise MSA is obtained from the IMGT/HLA database. The reads aligning to HLA regions are extracted **b** from the input BAM and they are realigned **c** to the sequences in the MSA. **d** A POG is constructed from MSA and further modified via alignment projection. **e** Haplotype assembly of two alleles is obtained by finding two paths (drawn in red and blue; overlap in purple) through the graph. DB database, HLA human leukocyte antigen, MSA multiple sequence alignment, POG partial-order graph, WGS whole-genome sequencing

Our method takes advantage of POGs to capture all known alleles and further modifies the graph to include variants found in the sequencing data so that the graph include the paths of true alleles. An overview of our method is illustrated in Fig. 2, and the major steps are labeled from (a) to (e). More details are given in “Methods”. We first create a comprehensive reference panel from a combined multiple sequence alignment (MSA) of both full-length and exon-only known alleles for each HLA locus (step a). Reads mapped to all known HLA loci in the human reference genome (GRCh38) are extracted (step b) and aligned to the comprehensive reference panel (step c). Gene-wise POGs are constructed using the combined MSAs. The alignments of the extracted reads are projected onto the graphs so that each read alignment is stored as a path in the graphs and the read depths on the edges naturally become edge weights (step d). When these read- or read-pair-backed paths connect two or more neighboring heterozygous sites of two alleles, they provide phasing information. During the alignment projection, the graphs are modified by adding nodes and edges to incorporate differences found by the alignment, such as substitutions and indels. Note that a sequence of an allele is encoded as a path through the entire graph. Finally, using the weighted graphs with alignment paths, we formulate the problem of constructing the best pair of HLA allele sequences as finding the pair of paths through the graph. When finding the pair, we consider consistent phasing information from the reads and coverage using base quality scores. Additionally, the pair of paths may be identical, to permit homozygous alleles.

### Simulation

To check that our method performs well, we tested it on simulated data (see “Methods” section). For each of the six HLA genes, two alleles from the set of full-length gene sequences in the IPD-IMGT/HLA database were randomly chosen. We repeated this for a total of 100 replicates, resulting in 200 randomly selected alleles across all replicates. For each replicate, we simulated 50 × coverage (25 × for each haplotype) of paired-end WGS data. We compared Kourami, PHLAT, and HLA*PRG on the simulated data. Our method was evaluated using all 1200 alleles (2 alleles × 6 genes × 100 replicates). However, not all alleles could be used for the evaluation of PHLAT and HLA*PRG, as both tools use their own digested format of the HLA database, which is built in, so that the content of the database cannot be updated by a user. The database versions used by PHLAT and HLA*PRG are older compared to the version (v3.24.0) used for Kourami. Given a set of WGS data of an individual with an allele that is not in the database built into PHLAT and HLA*PRG, both tools will fail to type the allele correctly as they are designed strictly to find the nearest match among the known alleles. For this reason, the evaluations of PHLAT and HLA*PRG are based only on the subset of simulated alleles (1011 for PHLAT and 990 for HLA*PRG) that are in the database versions they use. For PHLAT, four-digit P resolution was used and six-digit G resolution was used for HLA*PRG and Kourami for evaluation.

Table 1 shows the number of correctly inferred alleles as well as the accuracy for each HLA gene tested. For our method, we report both the typing and assembly accuracy. We define the assembly accuracy as the percentage of assembled alleles with sequence identical to the true allele sequence (no mismatch or indel). Even when an assembled allele is not identical to its expected true sequence, the typing of the allele may be correct if the closest sequence (minimum edit distance) in the database is the true allele. PHLAT achieves 93.6% accuracy across all HLA genes tested (89.8% for class I and 96.6% for class II). HLA*PRG and our method perform equally well, achieving 99.8% typing accuracy across all genes (99.5% for class I and 100% for class II). Additionally, Kourami achieves 99.3% assembly accuracy.

**Table 1:** HLA typing performance on simulated data

	Class I			Class II		
	A	B	C	DQA1	DQB1	DRB1
PHLAT (four-digit P)	0.85 (147/172)	0.91 (135/149)	0.95 (122/129)	0.93 (178/191)	0.98 (169/173)	0.99 (195/197)
HLA*PRG (six-digit G)	1.00 (174/174)	0.99 (143/144)	0.99 (115/116)	1.00 (197/197)	1.00 (159/159)	1.00 (200/200)
Kourami (type)	1.00 (199/200)	1.00 (200/200)	0.99 (198/200)	1.00 (200/200)	1.00 (200/200)	1.00 (200/200)
Kourami (sequence)	0.99 (198/200)	1.00 (200/200)	0.98 (195/200)	1.00 (200/200)	1.00 (199/200)	1.00 (200/200)

Accuracy is shown as a fraction. The fraction of the number of correctly typed alleles and the total number of alleles tested are shown in parentheses

### Novel allele detection

The major benefit of our method is that it can assemble novel alleles across the typing exons. Therefore, its typing ability is not limited by known alleles as is the case with other database-matching methods. Unlike these methods, Kourami uses the known alleles in the input database only to construct the HLA graph that serves as a template for the reference-based assembly and it does not discriminate between the paths that encode known alleles and novel alleles.

To demonstrate its ability to assemble novel alleles, we evaluated Kourami across various data for which the ground truth is known. We tested with simulated data and real data with previously validated HLA types (NA12878–NA12891–NA12892 Platinum trio and 11 samples from the 1000 Genomes Project) with a modified database of known alleles so that Kourami is not aware of the true allele sequences. For each sample, we randomly selected one allele from each of the six HLA genes and removed the selected alleles from the reference MSAs (full-length and exon-only) provided by release 3.24.0 of the IPD/IMGT-HLA database. When removing an allele, we removed all entries in the G group to which the allele belongs. The entire list of alleles removed from each individual is shown in Additional file 1: Tables S1, S2, and S3. We removed corresponding rows for the alleles from both the full-length MSA and exon-only MSA and obtained a new reference panel by combining the modified MSAs. The number of G group alleles removed is shown in Table 2. The extracted paired-end reads were aligned to the newly obtained reference panel, and the BAM files obtained were used as inputs to Kourami. Note that this experiment cannot be done with PHLAT and HLA*PRG, as the database of known alleles is built into the tools.

**Table 2:** Novel allele recovery

	Simulation	Platinum trio	1000 Genomes
Number of removed alleles	596	15	60
Number of recovered alleles	586	15	59
Percentage of recovered alleles	98.3	100	98.3

Kourami correctly assembled 98.3%, 100%, and 98.3% of the removed alleles for the simulation data, the Platinum trio, and 11 samples from the 1000 Genomes Project, respectively (Table 2). Among 1000 Genomes samples, the only incorrectly assembled allele (supposed to be B*38:01:01) had a 1-bp difference from the correct sequence. When the 59 correctly assembled allele sequences are aligned to the newly constructed reference panel, many alleles were aligned equally well to a large number of known alleles. For example, C*05:01:01 alleles aligned to 122 other alleles with just 1-bp substitution. Among them, a significant portion contained base differences that result in protein-coding changes in typing exons. This shows that the database-matching methods such as PHLAT and HLA*PRG cannot be relied upon to select a closely related allele sequence in the presence of novel alleles. The database-matching methods often provide quality metrics for inferred alleles. Whether such quality metrics are effective when novel alleles are involved is still an open question. Recently, this was explored by the authors of HLA*PRG, and it was shown that their metrics were not effective in distinguishing novel alleles [11].

### Illumina Platinum Genomes

#### Platinum trio with validated results

Among the Illumina Platinum Genomes, we first ran Kourami, PHLAT, and HLA*PRG on the trio (NA12891, NA12878, and NA12892) with the previously validated four-digit HLA types for six HLA genes (HLA-A, -B, -C, -DQA1, -DQB1, and -DRB1) [11, 12]. Kourami and HLA*PRG perfectly called the correct types whereas PHLAT missed a call in the HLA-C gene in NA12891. In a previously published article [11], PHLAT called all 12 alleles correctly and the difference may be because in our evaluation, all software was run on the set of reads that aligned to the extended MHC (xMHC)/HLA region of chromosome 6 and unmapped reads. Extracting a subset of reads by read mapping location and including unmapped reads are common for reducing the computational time, and a similar technique was used in [10].

#### Trio consistency and inferred haplotypes

**Fig. 3:**
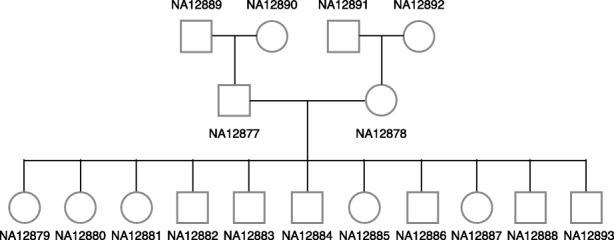
CEPH/Utah pedigree 1463. The family pedigree of Illumina Platinum Genomes is shown

The pedigree of Illumina Platinum Genomes includes many third-generation offspring and only the top right-hand trio in Fig. 3 has previously validated HLA typing results. Since this trio includes the mother (NA12878) of all third-generation offspring, if HLA typing results are trio-consistent across all trios and all second-generation haplotypes are present in one of the children, we can theoretically infer the HLA haplotypes of the second-generation male (NA12877) as well as the half of HLA haplotypes in the first-generation individuals (NA12889 and NA12890).

**Fig. 4:**
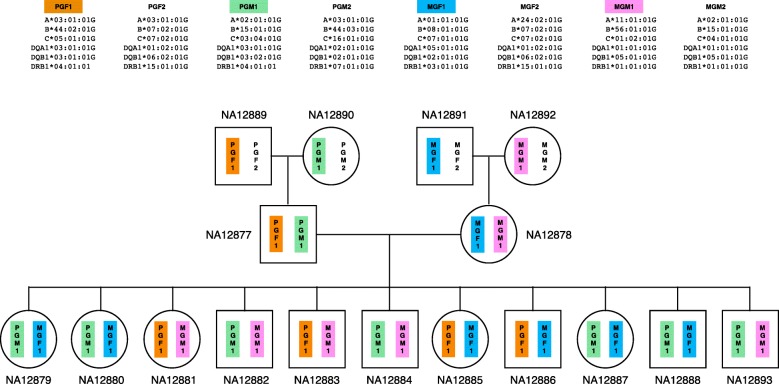
HLA haplotypes in the Illumina Platinum pedigree. Shown is the Illumina Platinum pedigree with the predicted HLA haplotype information. Four haplotypes (PGF1, PGM1, MGF1, and MGM1) of the second generation are intermixed in third-generation offspring. Only the haplotypes that are passed down to the second and third generations are colored. A haplotype drawn on the left is always inherited from the father. For the first generation, this information is missing and the haplotypes that are passed to the next generation are arbitrarily drawn on the left

We tested all three methods to determine whether predictions are trio-consistent across all trios (trio consistency is shown in Table 3). Kourami and HLA*PRG agreed on all 204 alleles at six-digit G resolution and the predicted alleles were trio-consistent. The inferred haplotypes across HLA genes (intra-gene phased) are shown in Fig. 4. PHLAT’s predictions were trio-consistent only for HLA-C and HLA-DQB1 when evaluated at four-digit P resolution, and additionally for HLA-A when evaluated at two-digit resolution. Although, we do not know the true HLA types for the rest of the 14 individuals, it is very likely that the predicted HLA types are correct given that all typing results are consistent. Low trio-consistency ratios for PHLAT in Table 3 are mainly due to mistyped alleles in HLA-A, HLA-B, and HLA-DRB1 for the NA12877 individual (which affects all 12 trios being evaluated). Trio-consistency measures the level of consistency rather than accuracy. PHLAT’s overall typing accuracy (four-digit) on all of the Platinum individuals (17 members) is 0.907, assuming the HLA types inferred by Kourami and HLA*PRG are true. Assuming the predicted HLA types for the pedigree are correct, no recombination seems to have occurred, leaving no disruption in ancestral haplotypes. In Fig. 4, we labeled the haplotypes that originated from the first-generation members as paternal grandfather 1 or 2 (PGF1 or PGF2), paternal grandmother 1 or 2 (PGM1 or PGM2), maternal grandfather 1 or 2 (MGF1 or, MGF2), and maternal grandmother 1 or 2 (MGM1 or MGM2). The haplotypes that are passed to second-generation individuals are numbered 1 to keep the numbering consistent in the third generation. Among 11 third-generation offspring, all four possible pairs of haplotypes were observed (two PGF+MGF, two PGF+MGM, four PGM+MGF, and three PGM+MGM).

**Table 3:** Trio consistency over 12 trios in Platinum Genomes

	Class I			Class II		
	A	B	C	DQA1	DQB1	DRB1
PHLAT (two-digit)	1.00 (24/24)	0.71 (17/24)	1.00 (24/24)	0.79 (19/24)	1.00 (24/24)	0.96 (23/24)
PHLAT (four-digit P)	0.67 (16/24)	0.42 (10/24)	1.00 (24/24)	0.79 (19/24)	1.00 (24/24)	0.46 (11/24)
HLA*PRG (six-digit G)	1.00 (24/24)	1.00 (24/24)	1.00 (24/24)	1.00 (24/24)	1.00 (24/24)	1.00 (24/24)
Kourami	1.00 (24/24)	1.00 (24/24)	1.00 (24/24)	1.00 (24/24)	1.00 (24/24)	1.00 (24/24)

Trio consistency is shown as a fraction. The number of consistent alleles is shown as a fraction in parentheses

### Genomes

We tested all three methods on this data set and the result is summarized in Table 4. PHLAT called 93 out of 122 alleles correctly, resulting in 76% accuracy when evaluated at four-digit P resolution, and 89% when evaluated at two-digit resolution. The results for HLA*PRG were consistent with what has previously been reported [11], resulting in one error (99.2% accuracy). Our method correctly called all of the alleles without any differences in bases. Note that the total number of alleles tested for DQA1 is 12 instead of 22 (2 alleles × 11 individuals) because the validation data for 1000 Genomes [32] does not report DQA1 types. DQA1 type validation is only available for six individuals [12].

**Table 4:** HLA typing performance on 11 individuals from the 1000 Genomes Project

	Class I			Class II		
	A	B	C	DQA1	DQB1	DRB1
PHLAT (two-digit)	0.82 (18/22)	0.82 (18/22)	0.91 (20/22)	0.83 (10/12)	1.00 (22/22)	0.95 (21/22)
PHLAT (four-digit P)	0.68 (15/22)	0.55 (12/22)	0.77 (17/22)	0.83 (10/12)	0.95 (21/22)	0.82 (18/22)
HLA*PRG (six-digit G)	1.00 (22/22)	1.00 (22/22)	1.00 (22/22)	1.00 (12/12)	1.00 (22/22)	0.95 (21/22)
Kourami (six-digit G)	1.00 (22/22)	1.00 (22/22)	1.00 (22/22)	1.00 (12/12)	1.00 (22/22)	1.00 (22/22)

Accuracy is shown as a fraction. The fraction of the number of correctly typed alleles and the total number of alleles tested are shown in parentheses

### CPU and memory usage

Kourami is able to assemble and type HLA alleles given WGS data in a fraction of the time compared to the state-of-art methods such as PHLAT and HLA*PRG with a moderate use of memory. We compared the CPU and memory usage using the WGS of NA12878 from Platinum Genomes data (2 × 101 bp, 55 ×). All tests were run on the input of the reads aligning to the xMHC region and unmapped reads. HLA*PRG was the slowest, taking 54.62 CPU hours, while PHLAT took 10.73 CPU hours and Kourami took only 0.09 CPU hours (611 × speedup compared to HLA*PRG). HLA*PRG required the most amount of memory, consuming peak memory of 78.9 Gbytes, while PHLAT and Kourami used 3.6 Gbytes and 4.3 Gbytes, respectively. HLA*PRG requires many more CPU hours and a larger amount of memory because of the expensive dynamic-programming-based alignment to the graph. Kourami relies on fast NGS aligners to align reads against known alleles first and projects the alignment obtained to the HLA graph to reduce the computational time significantly without sacrificing assembly or typing accuracy.

## Discussion

We have shown that our HLA assembly method can accurately reconstruct both haplotypes that span the typing exons of HLA genes using a graph representation of known alleles as a guide. The haplotype sequences produced can be used successfully for HLA typing given high coverage (>30-fold) paired-end WGS data. WGS carried out for another analysis can be used to type an individual’s HLA types without requiring another experiment (SBT or other molecular assays).

Notably, the ability to discover novel alleles from the highly accurate HLA assembly is achieved by using a flexible graph structure to represent all known alleles and allowing systematic modification to encode variants present in reads. This unique ability is instrumental in both research and clinical settings. Importantly, previously available computational methods using untargeted sequencing data cannot discover novel alleles because they are designed to find the best-matching allele among the known alleles.

The ability to discover novel alleles is especially beneficial when studying an understudied group of individuals harboring many novel alleles, such as African populations, which are known to exhibit a higher level of genetic diversity [33] compared to other ethnic populations. Disease pressure in a population causes a positive and balancing selection of HLA alleles, producing high diversity [34].

Especially with the continuously decreasing cost of obtaining a personal genome, personal WGS data will only become more widely available, and our method can deliver accurate HLA typing without additional experiments and cost. Also, Kourami is able to assemble and type at six-digit G resolution in a fraction of the time compared to other methods with a moderate amount of memory usage.

One limitation of our method is that it primarily supports high-coverage WGS as it needs enough reads to cover both haplotypes for each typing locus, and may not work as well on other NGS assays, such as WES or RNA-seq data. Since WES is being used widely, as the cost for WES is lower compared to WGS, it is useful to be able to type HLA genes using WES. To assess Kourami’s ability to work with WES data, we tested Kourami on 29 HapMap individuals [35] (Additional file 1: Table S4). It was able to assemble and type 284 alleles out of 345 alleles with previously confirmed types. Kourami did not output an assembled sequence or typing results for the other 61 alleles as some regions of these alleles were not covered by reads, causing the graph to be disconnected within the typing exons. Among the 284 alleles that were assembled, Kourami was able to assemble and type 269 alleles correctly, achieving 94.7% accuracy. Among the 15 erroneous calls, seven were called as homozygous alleles instead of heterozygous. Reference allele bias due to capture bias may be the direct cause for erroneous homozygous calls. It is a known bias in WES along with other biases, such as GC bias. Moreover, coverage fluctuations have been reported [36, 37]. Collectively, these can cause a decrease in effectiveness in detecting variants when using WES compared to WGS [37, 38].

Additionally, Kourami requires high-coverage WGS data to ensure accurate HLA assembly or typing. We randomly sampled coverages of 20 ×, 25 ×, 30 ×, 35 ×, and 40 × for five replicates from each of the Platinum Genomes and the 1000 Genomes, and tested Kourami on these samples. Its accuracy is shown in Additional file 1: Table S5. The accuracy was high at 35 × and 40 × coverages, being above 0.97 for both data sets. The accuracy stayed respectable down to 25 × coverage (0.94) for Platinum Genomes and down to 30 × for 1000 Genomes (0.94). At 20 × coverage, the accuracy for both data sets dropped below 0.90 (0.87 for Platinum and 0.83 for 1000 Genomes across the HLA genes). This should not be a surprise, as haplotype-resolved assemblies of human genomes used ≈100× coverage of NGS data [39, 40]. The database-matching state-of-the-art HLA*PRG was shown to be more stable, even at 20 × coverage [11], indicating that database-matching techniques are more sensitive when high-coverage sequencing data are not available. It is known that accurate assembly or genotyping using NGS data with short read lengths requires high sequencing coverage, especially for human genome resequencing [41, 42]. Overall, the 1000 Genomes samples had lower accuracy compared to the Platinum Genomes samples, resulting in the largest difference in accuracy at the lowest coverage setting of 20 × (Additional file 1: Table S5). Sample-specific accuracies across different coverage settings are shown in Additional file 1: Figure S1 (Platinum Genomes) and Additional file 2: Figure S2 (1000 Genomes). The larger dips in accuracy for 30 × and below coverage settings observed in the 1000 Genomes samples are largely contributed by the HG01112, NA19625, and NA20502 samples. The longer Illumina reads (2 × 250 bp) used in 1000 Genomes samples are known to be more error prone, exhibiting increasingly higher error rates towards the 3^′^ end of the reads [43, 44] and this may be why the accuracy is more sensitive to the changes in coverage for the 1000 Genomes data set.

One of the main reasons for the requirement of high-coverage WGS data by Kourami is because the lower the coverage, the higher the chance for an allele-specific path to have a region with a local drop in coverage, resulting in a prohibitively low number of reads covering the region. This can be difficult for assembly methods. To investigate the effect of a local drop in coverage on the accuracy of Kourami, we extracted the minimum depth across the entire allele path for each allele call and investigated the fraction of incorrect calls that occur at various minimum depths (Additional file 2: Figure S3 for Platinum Genomes and Additional file 2: Figure S4 for 1000 Genomes). As expected, a higher fraction of calls made at smaller minimum depth were incorrect. Minimum depths of 1 and 2 caused Kourami to make mistakes. With a coverage of 30 × or higher, the minimum depth rarely plunged to 1 or 2, allowing Kourami to assemble alleles accurately.

In a typical HLA typing experiment, only a few classical HLA genes are typed, even though there are other classical HLA genes as well as non-classical HLA genes, which have also been shown to be disease-associated [45, 46]. There may be clinical and research importance in typing other HLA genes in addition to the six classical genes that Kourami mainly assembles. Since Kourami’s framework is sufficiently general to run on the additional HLA loci, we tested Kourami on 11 additional HLA genes by looking at trio-consistency for the Platinum trio and the Yoruban trio from 1000 Genomes (Additional file 1: Table S6). For the Platinum trio, we found all of the typing results to be trio-consistent. However, there were four loci where the assembled sequences were not identical to the typed allele at the sequence level. For HLA-H, HLA-J, and HLA-L genes, the mismatches to the typed database alleles were consistent across the trio, indicating that there may be novel allele sequences for these loci. For HLA-F, the child (NA12878) had a 1-bp mismatch to the corresponding allele of her parents. For the Yoruban trio, we also found all of the typed alleles to be trio-consistent. Like the Platinum trio, HLA-J and HLA-L alleles across the trio had consistent sequences but there were mismatches to the closest alleles in the database. HLA-F had a discrepancy of a 1-bp mismatch between the child (NA19240) and the parental alleles.

To validate Kourami further, we typed three additional individuals from the 1000 Genomes Project that were not benchmarked in [11] and a Korean individual, AK1 [40]. We tested Kourami, HLA*PRG, and PHLAT on these additional data. For these 1000 Genomes samples, we tested five validated loci (HLA-A, -B, -C, -DQB1, and -DRB1). For AK1, we tested six loci (by adding locus HLA-DQA1). Out of 42 alleles tested, PHLAT did not give a typing result for HLA-C on AK1 data and achieved an accuracy of 0.88 (35/40). All 42 calls were correct for both Kourami and HLA*PRG. One of the HLA-B alleles of the HG00268 individual that Kourami assembled had a single-nucleotide difference, although the typing was correct. For the AK1 genome, Kourami and HLA*PRG typed all six alleles correctly for the three additional validated loci (HLA-DRB3, -DPA1, and -DPB1).

## Conclusion

The highly accurate results from Kourami signify the recent advances in handling genetic variation using graph structures to encode variations found in multiple reference genomes [23, 27–29]. Specifically, in Kourami, the minimal representation of a POG allows the consistent graph modification via alignment projection and this in turn enables the capture of novel alleles as paths through the graph. At the same time, this reduces computational time greatly without sacrificing accuracy. Such improvements are necessary when used in high-demand clinical settings, although it may take some time for WGS-based typing to be widely used. Our approach can also be extended as a general method of using graph structures to guide the reference-based assembly of high-diversity gene families.

## Methods

### Input alignment and extraction of HLA reads

Kourami takes alignment of WGS to the human genome as an input in BAM format. For many experiments used here, we used pre-computed alignments downloaded from the European Bioinformatics Institute and Google Cloud Platform. If there were missing alignment files, we followed the 1000 Genomes procedures (see the GRCh38DH alignment “readme” file available from the 1000 Genome FTP server) to align reads using BWA-kit v0.7.15 [47] and further processed the BAM files using other tools, such as BioBamBam [48] and GATK [13].

From the alignments, we extracted paired-end reads aligned to all known HLA loci in chromosome 6, alternate sequences of xMHC regions, and HLA sequences (the complete set of coordinates used is in Additional file 1: Table S7) included in the human reference genome (hs38DH packaged in BWA-kit v0.7.15). In the GRCh38 assembly, regions that exhibit sufficient variability are represented in the primary chromosomal sequence as well as the alternate sequence loci scaffolds.

### Known HLA alleles and construction of a comprehensive reference panel

Immuno Polymorphism Database (IPD) periodically updates known HLA alleles in the IPD-IMGT/HLA database [8]. IPD-IMGT/HLA Release 3.24.0 (April 2016) was used for all experiments here. A detailed breakdown of the numbers of alleles included in this release is shown in Table 5. The other methods compared here use earlier versions of the database because the content of the database is built into their software, and there is no way to update or swap their databases at the user level. Using a later version of the database does not give advantages as long as the earlier version also contains the true alleles of the individual tested.

**Table 5:** Number of known HLA alleles used

Genes		Full-length	Total
		(exonic + full-length)	
Class I	A	218	3399
	B	337	4242
	C	301	2950
Class II	DQA1	45	69
	DQB1	27	911
	DRB1	40	1883

Release 3.24.0

Full-length denotes the total of number full-length alleles in the release and total number includes the full-length alleles and the alleles with only exon sequences reported

Many alleles in the database have only partial sequences, often just covering the few exons responsible for the peptide-binding domain of HLA genes (Table 5). For this reason, the IPD provides a set of pre-computed MSAs of full-length alleles (*M*_gene_) and the coding regions (*M*_coding_) separately for each HLA gene. Like HLA*PRG [11], for each HLA gene, we combine these two MSAs by aligning them at corresponding columns to obtain a comprehensive reference panel of known alleles. This can help in recruiting reads that span intron–exon junctions. The combined MSA (*M*_panel_) has the same number of rows as *M*_coding_. The number of columns in *M*_panel_ is equal to the sum of the number of columns in *M*_coding_ and the number of intronic columns in *M*_gene_. For each row in *M*_coding_, if the allele for the row has a corresponding row in *M*_gene_, intronic columns are inserted into *M*_coding_, otherwise, intronic columns of the reference allele in *M*_gene_ are inserted.

Non-polymorphic HLA genes DQA2 and DQB2 are paralogous copies of DQA1 and DQB1. They are often regarded as being poorly polymorphic. In addition to the HLA genes that are included in the IPD-IMGT/HLA database, DQA2 and DQB2 were added to the reference panel as decoys to filter out reads that originated from them and aligned incorrectly to other class II genes. In our analysis, we noticed that reads from DQA2 or DQB2 can make the assembly of typing exons of class II genes difficult, as previously reported [16].

### HLA graph construction

**Fig. 5:**
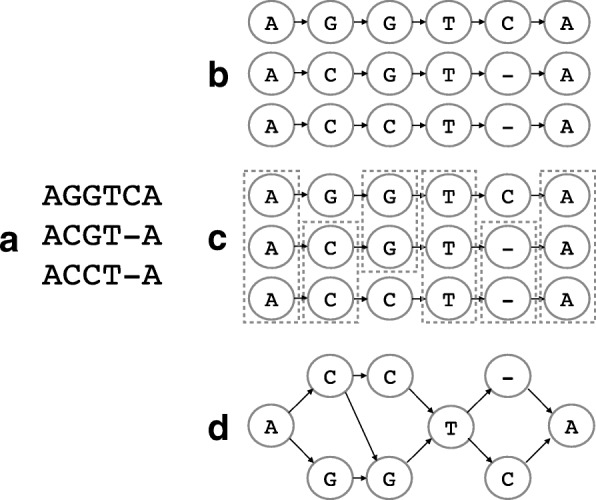
MSA to construction of a partial-order graph for HLA assembly. Given a pre-computed MSA (**a**), each sequence is constructed as a chain of vertices connected by directed edges. Corresponding positional vertices are aligned vertically (**b**). For each column, redundant vertices are grouped together, drawn within dotted boxes in (**c**). When they are merged, the corresponding partial-order graph (**d**) is obtained. HLA human leukocyte antigen, MSA multiple sequence alignment

To capture all information contained in *M*_panel_ in a minimal manner as well as to allow flexibility to enable novel sequence discovery, we use POGs, a compact graphical representation for MSA [24]. From each *M*_panel_, we can directly construct a gene-specific POG similar to those typically used in MSA [24, 49]. An example of a MSA of three known sequences (*M*_panel_) is shown in Fig. 5a. Each sequence is first drawn as a chain of vertices connected by directed edges (Fig. 5b), where each vertex *v*_*i*_ represents a base symbol $b_{v_{i}}$ ($b_{v_{i}} \in $ {A,C,G,T,N,-}) and is positioned at column *i* in the graph. For each column, vertices with an identical base symbol at a column are merged as a single vertex and duplicate edges are removed (Fig. 5c,d). The gap symbol (-) is used to restrict edges to connect vertices only from consecutive columns in the input MSA. An edge between two vertices ($e_{v_{i}, v_{i+1}}$) exists if *M*_panel_ has a row with consecutive bases $b_{v_{i}}$ and $b_{v_{i+1}}$ at columns *i* and *i*+1. Note that this graph contains at least the same number of paths as the number of rows in *M*_panel_ used to construct the graph. The graph often encodes a larger number of paths and this flexibility is the foundation that allows us to model this family of sequences and capture novel alleles. For example, a simple graph shown in Fig. 5dencodes all sequences in the given MSA as well as AGGT-A, ACGTCA, and ACCTCA. Each path through the constructed graph encodes a possible allele.

### Modification of the HLA graph via alignment projection

Consider an example novel allele sequence, AGCTCA. It is easy to see that there is no path encoding such an allele in the HLA graph shown in Fig. 5d. In this example, simply adding an edge from the vertex G at column 2 to the vertex C at column 3 is the only modification needed for the graph to include the path that encodes the novel allele. If a novel allele exists in the data, there must be sequencing reads that contain the differences the novel allele has compared to known alleles. Assuming the sequence divergence is small enough for pairwise alignment of the read and a known allele to capture the differences, we can obtain the novel variants. For this reason, we further modify the HLA graph to include additional paths that encode for novel alleles in a test individual. We achieve this by modifying the previously constructed HLA graph by projecting the alignments of the reads likely coming from HLA region to known HLA genes.

**Fig. 6:**
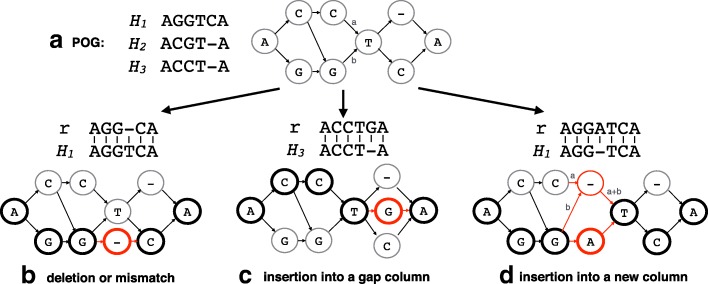
Modification by alignment projection. The multiple sequence alignment and its corresponding POG from Fig. 5 are shown (**a**). Three examples of graph modification operations (deletion or mismatch (**b**), insertion into a gap column (**c**), and insertion into a new column (**d**)) are shown with respect to the initial POG constructed. For each operation, an alignment of read *r* to one of the known alleles *H*_*i*_ is used to modify the graph. Each operation is applied to the POG and the resulting graph is shown. The nodes and edges that are newly added or changed during the operation are shown in red. The nodes that the read path maps are shown as bold circles. For an insertion into a new column, the newly assigned edge weights are explicitly drawn in using *x* and *y* variables. POG partial-ordered graph

We first align the extracted reads to the set of reference panel sequences obtained from *M*_panel_ using BWA (v0.7.15-r1140) [47]. The linear alignments obtained are then projected onto gene-specific POGs. For example, if a read is mapped to the HLA-A gene, then the alignment is projected onto the HLA graph of the gene. Given a read *r*, a subsequence *h* of a known allele *H*, and a pairwise alignment of *r* and *h*, by projection of the alignment to the HLA graph, our goals are: (1) to modify the graph to encode the exact sequence of *r* within the range of columns *h* encoded in the graph, (2) to increment the weight of each edge of the path by 1, and (3) to preserve preexisting paths at the same time. When *r* and *h* are identical, the graph must already contain a path that exactly encodes *r* because *H* is in the MSA used to construct the graph. When there are few differences identified by the pairwise alignment of *r* and *h*, such as mismatches, deletions, or insertions, there are two cases: (1) *r* is already encoded in the graph or (2) *r* is not, thereby the graph must be modified to encode *r*. For example, ACGTCA does not align perfectly to any of the sequences in Fig. 6a but it is encoded in the graph as a path. On the other hand, there is no path encoding ACCTGA.

Examples of graph modification by alignment projection are shown in Fig. 6. Panel (a) shows a MSA with three known alleles and the corresponding POG. Modifications for mismatches and deletions are simple because they require only the addition of a vertex for the mismatched base or a gap (-) symbol. Figure 6b illustrates an example where *r* has a deletion of T at position 4. A gap vertex is added to the corresponding column and edges are added to connect the newly added vertex to the previous and next base in *r* to obtain a path encoding *r*. Normally, an insertion requires a shifting of columns in the MSA and graph because extra columns are required for the inserted bases to be encoded. However, some alignments with insertions do not require a column shift. An example of an alignment with insertion not requiring a column shifting is shown in Fig. 6c. The read is aligned to *H*_3_ with an insertion at position 5 instead of aligning to an allele *H*_1_ with a mismatch at the same position because the alignment score with one insertion is higher than the score with three mismatches (positions 2, 3, and 5 if aligned to *H*_1_). Because *H*_1_ is in the MSA, the graph already has the column for handling an insertion at this particular column. In this case, we simply insert a vertex of G into the corresponding column and connect the edges to complete the path for *r*.

Finally, an insertion requiring a shift of columns is depicted as an example in Fig. 6d. The read is aligned to allele *H*_1_ with an insertion of A at position 4. To insert a new column between the third and fourth columns (also denoted as the left and right columns), we first insert a new vertex with a - symbol and need to reroute all edges between the left and right columns through the newly inserted gap symbol and redistribute edge weights to preserve the preexisting paths. Adjusted weights are shown on the edges in the example. To describe this formally, let *L* and *R* be sets of vertices for the left and right columns respectively and *E* be the set of directed edges from *v*_*l*_∈*L* to *v*_*r*_∈*R* with the weight of each edge as *w*({*v*_*l*_,*v*_*r*_}). Additionally, let $E^{\text {out}}_{v_{l}}\subseteq E$ be the set of all outgoing edges of *v*_*l*_ and $E^{\text {in}}_{v_{r}}\subseteq E$ be the set of all incoming edges of *v*_*r*_. Note that there are always one or more outgoing edges from *v*_*l*_ and one or more incoming edges to *v*_*r*_. After disconnecting all {*v*_*l*_,*v*_*r*_}∈*E*, we make a new vertex *v*_gap_ with the - symbol and add an edge {*v*_*l*_,*v*_gap_} for each *v*_*l*_ and assign a weight of $\sum _{e\in E^{\text {out}}_{v_{l}}}w(e)$. Similarly, we add an edge {*v*_gap_,*v*_*r*_} for each *v*_*r*_ with a weight of $\sum _{e\in E^{\text {in}}_{v_{r}}}w(e)$. Once the column shift is done, we can actually process the insertion of the base exactly as we handled the insertion into a gap column.

### Finding paths through the HLA graph

Given an HLA graph with weights, assembling HLA alleles can be formulated as the problem of finding two (diploid) paths (they can be identical) that explain the read mapping data (weights and phasing) best. When considering only the weights (number of reads), we can find two paths where the sum of their minimum weights is maximized. However, this formulation does not handle phasing information embedded by reads or read pairs. Therefore, it can possibly select erroneous paths that are not consistent with the phasing information. For this reason, we want our objective to take both weights as well as phasing information into account. Since read information is embedded in the HLA graph, we can check if two neighboring variant sites can be phased directly by a read or read pair. For example, given two heterozygous sites with A/T and G/C, a read or a read pair carrying A followed by G at these sites indicates the chromosomal phase of AG since the sequencing read is assumed to come from a contiguous segment in a chromosome. In our method, we first investigate variant regions individually to select locally phased paths with strong read support and construct a set of full-length paths through the HLA graph by connecting the locally phased paths that can be further phased by a read or read pair. Each of these full-length paths is considered as a candidate allele and the best pair among the candidates with maximum read and phasing support is selected as the output. To consider only nonzero-weight full-length paths, we remove all zero-weight edges and disconnected vertices prior to finding paths.

#### HLA graph to bubble graph

**Fig. 7:**
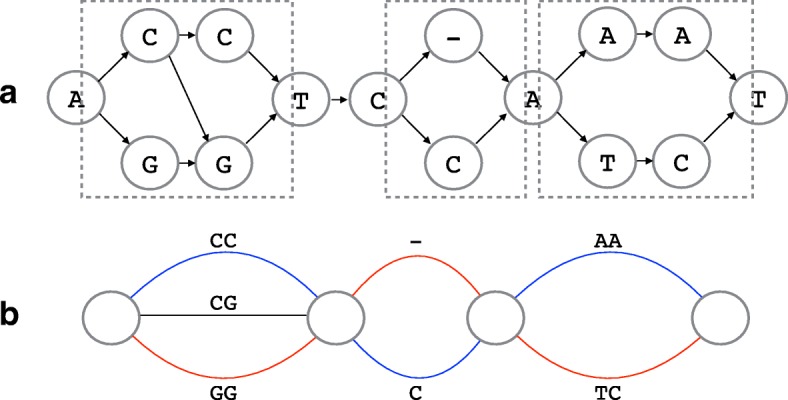
HLA graph to bubble graph. Show is an example of an HLA graph with three bubbles (in dotted boxes) (**a**) and its corresponding bubble graph (**b**). The best paths through the bubbles can be thought of as a pair of distinct colored paths (shown in red and blue). HLA human leukocyte antigen

We first focus on the parts of the HLA graph where variations are captured, which are often referred to as bubbles in sequence assembly graphs [50–53]. In a HLA graph, we define a *bubble* as a region (three or more consecutive columns) where there is only one vertex each in the leftmost and rightmost columns and the rest of the columns must have two or more vertices. Let *s* and *t* be the vertices in the leftmost and the rightmost columns of the bubble, respectively. Any vertex in the bubble is reachable from *s* and one or more paths exists from any vertex in the bubble to *t*. Any two distinct paths that go through a bubble must go through *s* and *t*. Bubbles naturally capture the variation of sites between two alleles in the graph. The regions that are enclosed by the dotted line in Fig. 7a are examples of bubbles. On the other hand, a region that is completely shared by all paths through the HLA graph represents a conserved region. Without any loss of generality, the HLA graph can then be thought of as a chain of bubbles, where two neighboring bubbles are connected by a linear path of length 0 or longer (Fig. 7). For simplicity, we can connect the bubbles without the linear paths as they do not play any role in determining the phase of a haplotype. We call this a bubble graph. Bubbles can easily be recognized in a HLA graph because of its structure.

#### Finding the best set of paths in a bubble

Ideally, we want to find exactly two paths per bubble since the ploidy number is two for humans. The paths can be identical for homozygous alleles. However, bubbles may contain more than two paths because of sequencing errors or misalignment. Therefore, we first identify all paths that are phased by a read or read pair. For each bubble, we can use a modified breadth-first search technique to obtain all paths that go through the bubble. To avoid enumerating over all paths through a bubble, we prune any path without a read backing the sequence encoded by the path at each iteration of the breadth-first search. For a path in the bubble to be retained, it must be supported by at least one read phasing the entire path. We can simply compute the set of phased reads for a path by taking a series of intersections of read sets maintained by each edge in the path. Each phased path through a bubble is a called a *bubble path*.

Given multiple bubble paths from a bubble, our goal is to select the best pair of paths. We iterate over all possible pairs of bubble paths to calculate the posterior probability of each pair given all reads aligned to the bubble to find the pair that gives the maximum probability. We write the posterior probability of a given genotype as

$$P(G_{b}\mid D)=\frac{P(G_{b})P(D\mid G_{b})}{P(D)}, $$

where *G*_*b*_ is a genotype and *D* is the alignments of all reads aligned over the bubble. The genotype is a pair of bubble paths *G*_*b*_=(*H*_*b*1_,*H*_*b*2_). Each *d*∈*D* is an alignment string of a segment of a read and *d*^*i*^ is the *i*th symbol in segment *d*. Similarly, $H_{b1}^{i}$ is the *i*th symbol in *H*_*b*1_. *P*(*D*) is constant, and we assume that the prior probability *P*(*G*_*b*_=(*H*_*b*1_,*H*_*b*2_)) is uniformly distributed over all genotypes. We can then compute the conditional probability *P*(*D*∣*G*_*b*_) by adopting widely used formulations [13, 54] with small variations to allow multiple positions and the base N case that can be present from sequence data. We iterate over each read and compute *P*(*D*∣*G*_*b*_) as a product of the conditional probability of each read *d*.

Since a read must come from one of the two chromosomes, and we assume that *d* is equally likely to come from either one of them, we can rewrite it as a sum of the average of two cases where *d* is from *H*_*b*1_ and *H*_*b*2_:

$$P(D\mid G_{b}) = \prod\limits_{d\in D}{\left[\frac{1}{2}P(d\mid H_{b1}) + \frac{1}{2}P(d\mid H_{b2})\right]}. $$

To compute the conditional probability of each *d* given a bubble path *H*_*b*_, we iterate over each pair of corresponding positions *d*^*i*^ and $H_{b}^{i}$ jointly, assuming each *d*^*i*^ is conditionally independent of each other given $H_{b}^{i}$. Therefore, the probability of each base *d*^*i*^ given a pair of corresponding genotype bases $H_{b1}^{i}$ and $H_{b2}^{i}$ is

$$\frac{1}{2}P \left(d^{i}\mid H_{b1}^{i} \right) + \frac{1}{2}P \left(d^{i}\mid H_{b2}^{i}\right). $$

The probability of seeing a base given an allele is defined as

$$\begin{array}{*{20}l} P\left(d^{i}\mid H_{b}^{i}\right) &= \left\{ \begin{array}{ll} 1-\epsilon, & \text{if } d^{i}=H_{b}^{i} \text{~~~(match)} \\ \epsilon/3, & \text{if } d^{i}\neq H_{b}^{i} \text{~~~(mismatch)} \end{array}\right. {,} \end{array} $$

where *ε* of base symbol *d*^*i*^ is the error probability obtained from the phred score of the base. For *d*^*i*^= N, we simply estimate the probability as 1/4. Instead of selecting *H*_*b*_ from all possible |*d*|-mers, we limit to only the bubble paths found in the bubble and iterate over all pairs to select a pair of bubble paths $\mathcal {P}_{b}$ that jointly gives the maximum probability:

$$\mathcal{P}_{b} = \text{argmax}_{G_{b}} \prod\limits_{d\in D}\prod_{i}^{|d|} \left[ \frac{P\left(d^{i}\mid H_{b1}^{i}\right)}{2} + \frac{P\left(d^{i}\mid H_{b2}^{i}\right)}{2} \right]. $$

#### Phasing paths

We now have an ordered list of bubbles, and a list of the best read-backed phased bubble paths for each bubble. The goal here is to find a set of candidate paths through all the bubbles by merging one bubble at a time iteratively from left to right, connecting bubble paths that are phased by a read or read pair. Two paths are said to be phase-consistent if there is a read or read pair spanning both paths. This can be checked easily by taking an intersection, since each bubble path maintains a set of phasing reads. Given a set of already merged bubble paths $\mathcal {P}_{m}$ from the first *i*−1 bubbles and a set of bubble paths $\mathcal {P}_{b_{i}}$ from the *i*th bubble, we look at all pairs of paths $\mathcal {P}_{m} \times \mathcal {P}_{b_{i}}$ and keep only pairs that are phase-consistent and connect each of such pairs as one path. We also update the phasing-read set for each merged path.

#### Selecting the best pair of candidate alleles

Once the assembly by bubble merging is finished, we have a set of merged bubble paths through all bubbles. By placing the linear chains that were ignored during bubble merging back to their original positions (between bubbles), we have a full-length candidate allele *H*_*i*_ for each merged bubble path. Let *C* be the set of all candidate alleles and *B* be a set of all bubbles. Our goal is to select a pair of alleles (*H*_1_,*H*_2_)∈*C*×*C* that has the most consistent phasing support over all bubbles. We first define a scoring metric that checks for the strength of phasing support jointly for a pair of alleles *H*_1_ and *H*_2_ between a pair of consecutive bubbles *b*_*i*_ and *b*_*i*+1_. It is defined as

$$ F_{i}(H_{1},H_{2}) =\left\{ \begin{array}{ll} f_{i}(H_{1})\times f_{i}(H_{2}), &\text{if } H_{1} \neq H_{2} \\ \frac{f_{i}(H_{1})}{2}\times \frac{f_{i}(H_{2})}{2}, & \text{if } H_{1} = H_{2} \end{array}\right. {,} $$

where *f*_*i*_(*H*) is the inter-bubble phasing fraction. This fraction *f*_*i*_(*H*) is the ratio of the number of phasing reads for allele *H* between *b*_*i*_ and *b*_*i*+1_ and the number of total phasing reads. When considering two paths at the same time, there can be regions where the paths overlap (homozygous; shown as purple edges in Fig. 2e) and separate (heterozygous; shown as blue or red edges in Fig. 2e). For homozygous sections of the paths, that is *H*_1_=*H*_2_, *f*_*i*_(*H*) is halved to keep the balance between calling homozygous and heterozygous alleles. We can calculate the product of *F*_*i*_ over all pairs of neighboring bubbles to check the consistency of phasing support for the pair. Finally, we select the pair of alleles $\mathcal {P}$ that maximizes the product over all pairs of alleles:

$$\mathcal{P} =\text{argmax}_{\{H_{1},H_{2}\}\in C \times C} \left[\prod\limits_{i}^{|B|-1}{F_{i}(H_{1},H_{2})}\right]. $$

### Description of data used for evaluation

#### Simulated data

For each of the six HLA genes tested (HLA-A, -B, -C, -DQA1, -DQB1, and -DRB1), we randomly selected two full-length alleles from the IPD-IMGT/HLA database (v3.24.0) and repeated this for 100 replicates, resulting in a total of 200 alleles to simulate. The exact number of full-length alleles in the database is reported in Table 5. For each replicate, we simulated 25 × coverage of paired-end WGS data for each allele, giving 50 × coverage for each locus. For the simulation of paired-end reads, we used an Illumina read simulator, pIRS [55], which simulates using empirical base-calling and GC%-depth profiles trained from Illumina re-sequencing of known samples. We used 2 × 100 bp for the read length and 500 ± 50 bp for the mean and the standard deviation of the insert size.

#### Illumina Platinum Genomes

Illumina has sequenced 17 individuals (CEPH/Utah pedigree 1463) in a three-generation family using their high-coverage PCR-free paired-end WGS assay (2 × 101 bp). These genomes are often referred to as the Illumina Platinum Genomes [56]. The family pedigree is shown in Fig. 3. Many individuals in this family have been extensively investigated by the genomics community, especially the NA12891–NA12892–NA12878 trio as well as the NA12889–NA12890–NA12877 trio. The validated HLA types for the two trios were obtained from [11, 32]. The read alignments to the GRCh37 version of the human genome for all 17 individuals were downloaded from the Illumina Platinum Genomes page hosted on Google Cloud Platform (Additional file 1: Table S8) and they were realigned to the GRCh38 version of the human genome.

#### Genomes

The 1000 Genomes Project [57] has produced various personal genomic data. Among these, there are 11 individuals used in [11] whose high-coverage WGS data along with validated HLA typing results [11, 32] are available. This data set covers a wide ethnic diversity (one Colombian from Medellín, three Utah residents with Northern and Western European ancestry in a trio, one Japanese from Tokyo, Japan, three Yoruban from Ibadan, Nigeria in a trio, one person of African ancestry from the southwestern United States, one person of Mexican ancestry from Los Angeles, and one Toscani from Italy) covering various different HLA types, making it an ideal data set to test on. The BAM files aligned to the GRCh38 version of the human genome were downloaded from the 1000 Genomes data portal (http://www.internationalgenome.org/data-portal). For the Utah resident trio and the Yoruban trio, we downloaded fastq files and realigned to the GRCh38 version because GRCh38 BAM files were not available. There are three additional individuals with validated HLA typing results [32] whose high-coverage WGS data are available. Pre-aligned BAM files of the additional individuals were downloaded.

#### Whole-exome sequencing of 29 HapMap individuals

Altogether, 29 HapMap [35] samples (WES) that were used as a benchmarking data set for HLA*PRG [11] were selected to test Kourami’s ability to assemble and type WES data. The 1000 Genomes data portal provides the BAM/CRAM files aligned to the GRCh38 version of the human genome for this set of WES data. The complete list of individual identifiers and the sources where the data were downloaded from are given in Additional file 1: Table S8.

#### AK1 Korean genome

De novo assembly of the Korean individual AK1 has been published [40]. Both the validated HLA types (Supplementary Table 20 in [40]) and high-coverage WGS data (2 × 150 bp) are publicly available. We downloaded fastq files and realigned to the GRCh38 version of the human genome. There is a link to the downloaded data in Additional file 1: Table S8.

## Additional files

Additional file 1 Supplementary tables with descriptions. (XLSX 70 kb)

Additional file 2 Supplementary figures with descriptions. (PDF 84 kb)

## References

[1] Sollid LM, Pos W, Wucherpfennig KW (2014). Molecular mechanisms for contribution of MHC molecules to autoimmune diseases. Curr Opin Immunol.

[2] Miyadera H, Tokunaga K (2015). Associations of human leukocyte antigens with autoimmune diseases: challenges in identifying the mechanism. J Hum Genet.

[3] Simmonds M, Gough S (2007). The HLA region and autoimmune disease: associations and mechanisms of action. Curr Genom.

[4] Matzaraki V, Kumar V, Wijmenga C, Zhernakova A (2017). The MHC locus and genetic susceptibility to autoimmune and infectious diseases. Genome Biol.

[5] Hedrick PW, Thomson G (1983). Evidence for balancing selection at HLA. Genetics.

[6] Black FL, Hedrick PW (1997). Strong balancing selection at HLA loci: evidence from segregation in South Amerindian families. Proc Natl Acad Sci USA.

[7] Ferrer A, Fernández ME, Nazabal M (2005). Overview on HLA and DNA typing methods. Biotecnología Aplicada.

[8] Robinson J, Halliwell JA, Hayhurst JD, Flicek P, Parham P, Marsh SGE (2015). The IPD and IMGT/HLA database: allele variant databases. Nucleic Acids Res.

[9] Major E, Rigó K, Hague T, Bérces A, Juhos S (2013). HLA typing from 1000 Genomes whole genome and whole exome Illumina data. PLoS ONE.

[10] Bauer DC, Zadoorian A, Wilson LO, Thorne NP, et al.Evaluation of computational programs to predict HLA genotypes from genomic sequencing data. Brief Bioinform. 2016. 10.1093/bib/bbw097. 10.1093/bib/bbw097 PMC6019030 27802932

[11] Dilthey AT, Gourraud PA, Mentzer AJ, Cereb N, Iqbal Z, McVean G (2016). High-accuracy HLA type inference from whole-genome sequencing data using population reference graphs. PLoS Comput Biol.

[12] Erlich RL, Jia X, Anderson S, Banks E, Gao X, Carrington M (2011). Next-generation sequencing for HLA typing of class I loci. BMC Genomics.

[13] McKenna A, Hanna M, Banks E, Sivachenko A, Cibulskis K, Kernytsky A (2010). The Genome Analysis Toolkit: a MapReduce framework for analyzing next-generation DNA sequencing data. Genome Res.

[14] Boegel S, Löwer M, Schäfer M, Bukur T, De Graaf J, Boisguérin V (2012). HLA typing from RNA-seq sequence reads. Genome Med.

[15] Kim HJ, Pourmand N (2013). HLA haplotyping from RNA-seq data using hierarchical read weighting. PLoS ONE.

[16] Bai Y, Ni M, Cooper B, Wei Y, Fury W (2014). Inference of high resolution HLA types using genome-wide RNA or DNA sequencing reads. BMC Genomics.

[17] Nariai N, Kojima K, Saito S, Mimori T, Sato Y, Kawai Y (2015). HLA-VBSeq: accurate HLA typing at full resolution from whole-genome sequencing data. BMC Genomics.

[18] Nariai N, Hirose O, Kojima K, Nagasaki M (2013). TIGAR: transcript isoform abundance estimation method with gapped alignment of RNA-seq data by variational Bayesian inference. Bioinformatics.

[19] Szolek A, Schubert B, Mohr C, Sturm M, Feldhahn M, Kohlbacher O (2014). OptiType: precision HLA typing from next-generation sequencing data. Bioinformatics.

[20] Xie C, Yeo ZX, Wong M, Piper J, Long T, Kirkness EF (2017). Fast and accurate HLA typing from short-read next-generation sequence data with xHLA. Proc Natl Acad Sci USA.

[21] Warren RL, Choe G, Freeman DJ, Castellarin M, Munro S, Moore R (2012). Derivation of HLA types from shotgun sequence datasets. Genome Med.

[22] Huang Y, Yang J, Ying D, Zhang Y, Shotelersuk V, Hirankarn N (2015). HLAreporter: a tool for HLA typing from next generation sequencing data. Genome Med.

[23] Dilthey A, Cox C, Iqbal Z, Nelson MR, McVean G (2015). Improved genome inference in the MHC using a population reference graph. Nat Genet.

[24] Lee C, Grasso C, Sharlow MF (2002). Multiple sequence alignment using partial order graphs. Bioinformatics.

[25] Pevzner PA, Tang H, Waterman MS (2001). An Eulerian path approach to DNA fragment assembly. Proc Natl Acad Sci USA.

[26] Myers EW (2005). The fragment assembly string graph. Bioinformatics.

[27] Paten B, Novak A, Haussler D. Mapping to a reference genome structure. arXiv.2014;1404.5010v1.

[28] Nguyen N, Hickey G, Zerbino DR, Raney B, Earl D, Armstrong J (2015). Building a pan-genome reference for a population. J Comput Biol.

[29] Church DM, Schneider VA, Steinberg KM, Schatz MC, Quinlan AR, Chin CS (2015). Extending reference assembly models. Genome Biol.

[30] Paten B, Novak AM, Eizenga JM, Garrison E (2017). Genome graphs and the evolution of genome inference. Genome Res.

[31] Marsh SGE, Albert ED, Bodmer WF, Bontrop RE, Dupont B, Erlich HA (2010). Nomenclature for factors of the HLA system, 2010. Tissue Antigens.

[32] Gourraud PA, Khankhanian P, Cereb N, Yang SY, Feolo M, Maiers M (2014). HLA diversity in the 1000 Genomes dataset. PLoS ONE.

[33] Campbell MC, Tishkoff SA (2008). African genetic diversity: implications for human demographic history, modern human origins, and complex disease mapping. Annu Rev Genomics Hum Genet.

[34] Prugnolle F, Manica A, Charpentier M, Guégan JF, Guernier V, Balloux F (2005). Pathogen-driven selection and worldwide HLA class I diversity. Curr Biol.

[35] International HapMap Consortium (2005). A haplotype map of the human genome. Nature.

[36] Xu Y, Jiang H, Tyler-Smith C, Xue Y, Jiang T, Asan (2011). Comprehensive comparison of three commercial human whole-exome capture platforms. Genome Biol.

[37] Meienberg J, Bruggmann R, Oexle K, Matyas G (2016). Clinical sequencing: is WGS the better WES?. Hum Genet.

[38] Belkadi A, Bolze A, Itan Y, Cobat A, Vincent QB, Antipenko A (2015). Whole-genome sequencing is more powerful than whole-exome sequencing for detecting exome variants. Proc Natl Acad Sci USA.

[39] Cao H, Wu H, Luo R, Huang S, Sun Y, Tong X (2015). De novo assembly of a haplotype-resolved human genome. Nat Biotechnol.

[40] Seo JS, Rhie A, Kim J, Lee S, Sohn MH, Kim CU (2016). De novo assembly and phasing of a Korean human genome. Nature.

[41] Ajay SS, Parker SC, Abaan HO, Fajardo KVF, Margulies EH (2011). Accurate and comprehensive sequencing of personal genomes. Genome Res.

[42] Ekblom R, Wolf JB (2014). A field guide to whole-genome sequencing, assembly and annotation. Evol Appl.

[43] Sameith K, Roscito JG, Hiller M (2017). Iterative error correction of long sequencing reads maximizes accuracy and improves contig assembly. Brief Bioinform.

[44] Schirmer M, DAmore R, Ijaz UZ, Hall N, Quince C (2016). Illumina error profiles: resolving fine-scale variation in metagenomic sequencing data. BMC Bioinform.

[45] Lefebvre S, Antoine M, Uzan S, McMaster M, Dausset J, Carosella ED (2002). Specific activation of the non-classical class I histocompatibility HLA-G antigen and expression of the ILT2 inhibitory receptor in human breast cancer. J Pathol.

[46] Bukur J, Jasinski S, Seliger B (2012). The role of classical and non-classical HLA class I antigens in human tumors. Semin Cancer Biol.

[47] Li H, Durbin R (2009). Fast and accurate short read alignment with Burrows–Wheeler transform. Bioinformatics.

[48] Tischler G, Leonard S (2014). biobambam: Tools for read pair collation based algorithms on BAM files. Source Code Biol Med.

[49] Löytynoja A, Vilella AJ, Goldman N (2012). Accurate extension of multiple sequence alignments using a phylogeny-aware graph algorithm. Bioinformatics.

[50] Fasulo D, Halpern A, Dew I, Mobarry C (2002). Efficiently detecting polymorphisms during the fragment assembly process. Bioinformatics.

[51] Iqbal Z, Caccamo M, Turner I, Flicek P, McVean G (2012). De novo assembly and genotyping of variants using colored de Bruijn graphs. Nat Genet.

[52] Sacomoto GA, Kielbassa J, Chikhi R, Uricaru R, Antoniou P, Sagot MF (2012). KISSPLICE: de-novo calling alternative splicing events from RNA-seq data. BMC Bioinform.

[53] Nijkamp JF, Pop M, Reinders MJ, de Ridder D (2013). Exploring variation-aware contig graphs for (comparative) metagenomics using MaryGold. Bioinformatics.

[54] Li H (2011). A statistical framework for SNP calling, mutation discovery, association mapping and population genetical parameter estimation from sequencing data. Bioinformatics.

[55] Hu X, Yuan J, Shi Y, Lu J, Liu B, Li Z (2012). pIRS: Profile-based Illumina pair-end reads simulator. Bioinformatics.

[56] Eberle MA, Fritzilas E, Krusche P, Källberg M, Moore BL, Bekritsky MA (2017). A reference data set of 5.4 million phased human variants validated by genetic inheritance from sequencing a three-generation 17-member pedigree. Genome Res.

[57] The 1000 Genomes Project Consortium (2015). A global reference for human genetic variation. Nature.

[58] Illumina Cambridge Ltd. Whole genome sequencing and variant calls for the Coriell CEPH/UTAH 1463 family. The European Nucleotide Archive. 2012. https://www.ebi.ac.uk/ena/data/view/PRJEB3381.

[59] IGSR: The International Genome Sample Resource data portal. http://www.internationalgenome.org/data-portal.

[60] Seo JS, Rhie A, Kim J, Lee S, Sohn MH, Kim CU, et al. *Homo sapiens*, AK1 genome sequencing and de novo assembly of an Asian individual. NCBI Short Read Archive. 2016. https://trace.ncbi.nlm.nih.gov/Traces/sra/sra.cgi?study=SRP068953.

[61] Lee H. Kourami: graph-guided HLA assembler. 2017. 10.5281/zenodo.1122533.

