# Supplementary material for: Kourami: graph-guided assembly for novel human leukocyte antigen allele discovery
Source: Genome Biol. 2018 Feb 7;19:16. doi: 10.1186/s13059-018-1388-2 (PMC5804087; doi:10.1186/s13059-018-1388-2)
Supplement: Supplementary file 2 — Supplementary figures with descriptions. (PDF 84 kb) [file 13059_2018_1388_MOESM2_ESM.pdf]

SUPPLEMENTARY FIGURES

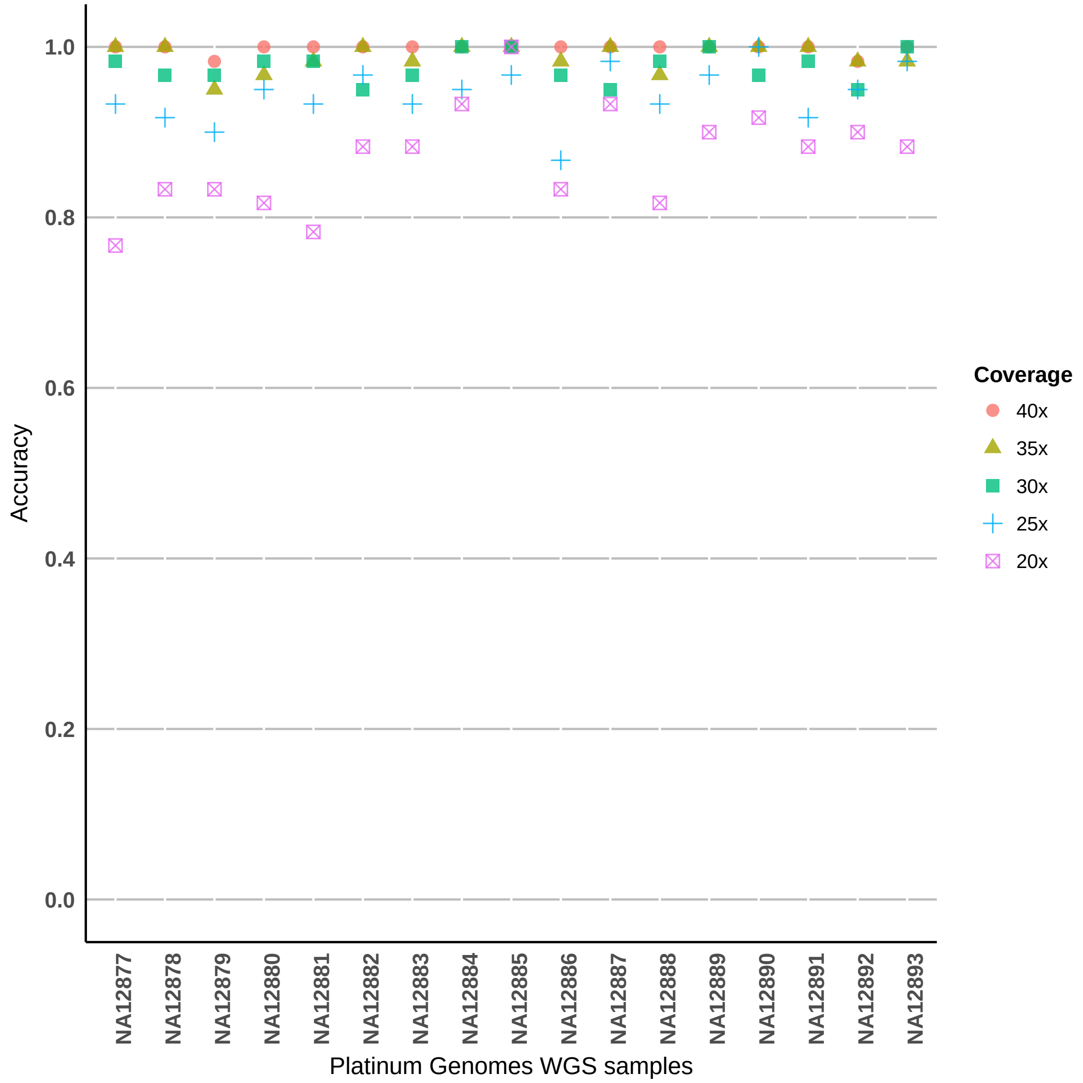

Figure S1. Sample-specific typing accuracy of `Kourami` across varying coverages for 17 Platinum Genomes. Each data point represents the fraction of correct calls across 5 replicates. The total number of calls is 60 (5 replicates $\times$ 12 calls) per data point.

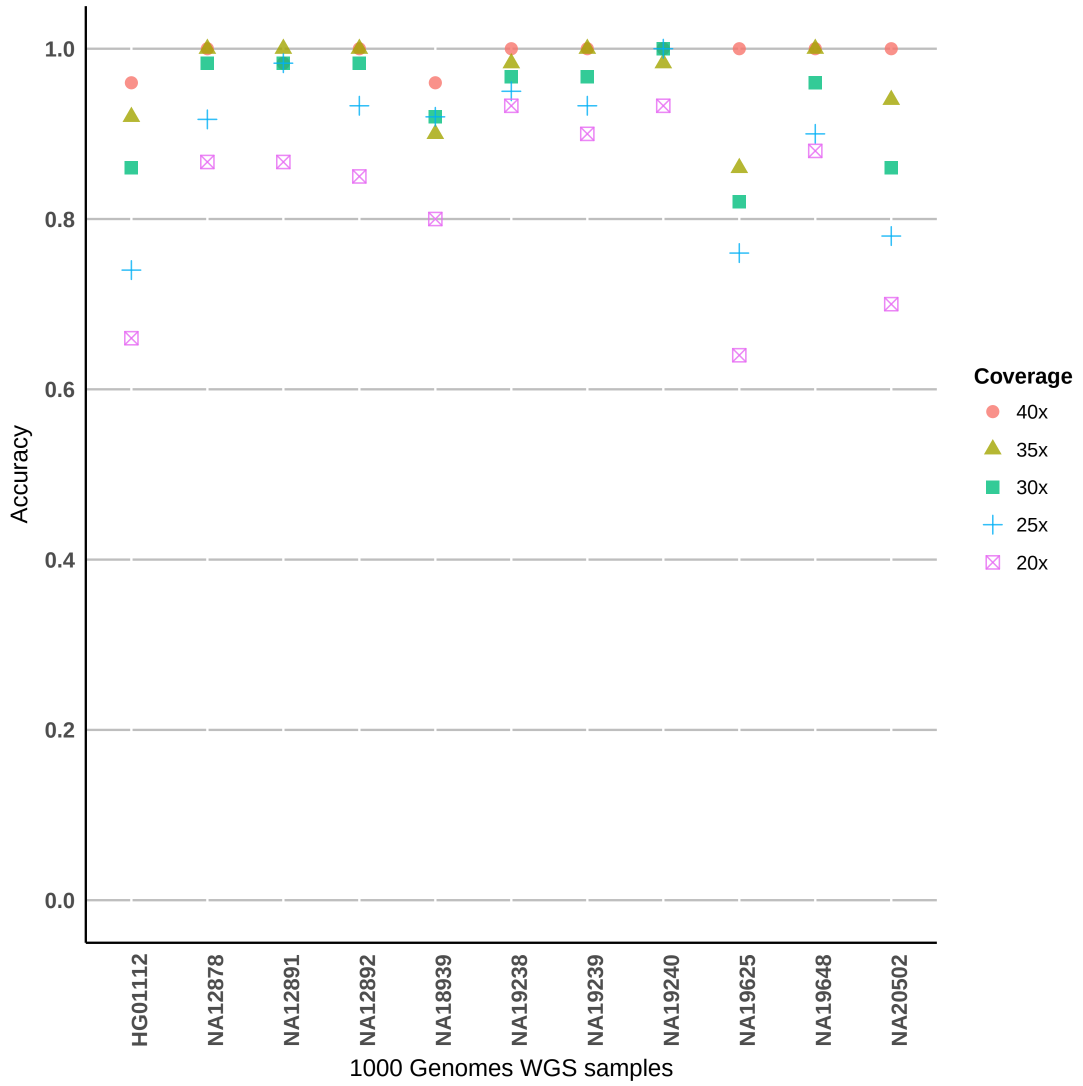

Figure S2. Sample-specific typing accuracy of `Kourami` across varying coverages for 11 samples from 1000 Genomes. Each data point represents the fraction of correct calls across 5 replicates. The total number of calls is 60 (5 replicates $\times$ 12 calls) for the Utah resident trio and 50 (5 replicates $\times$ 10 calls) for the Yoruban trio per data point.

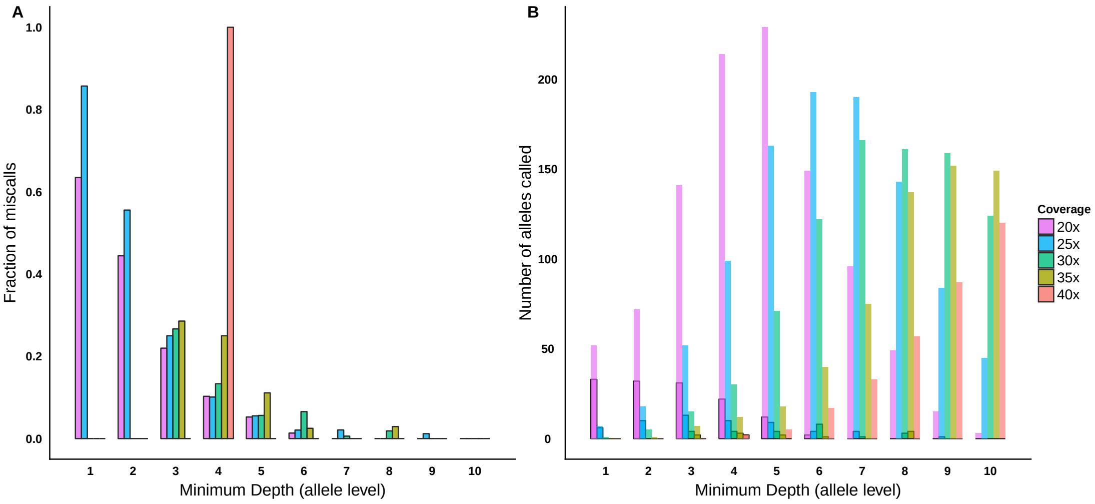

Figure S3. Incorrect calls at allele-specific minimum depths across varying coverages for 17 Platinum Genomes. Panel A shows the fraction of incorrect calls at varying allele-specific minimum depths across varying coverages. Panel B shows the total number of incorrect calls (black border) in front of the total number of alleles (no border, lighter shade) called at varying allele-specific minimum depths across varying coverages. The high peak at 40x coverage for minimum depth of 4 is caused by the small number of total calls made (see the corresponding bar on Panel B). Minimum depths on the x-axis are only shown up to 10 as the calls with larger minimum depths are all correct.

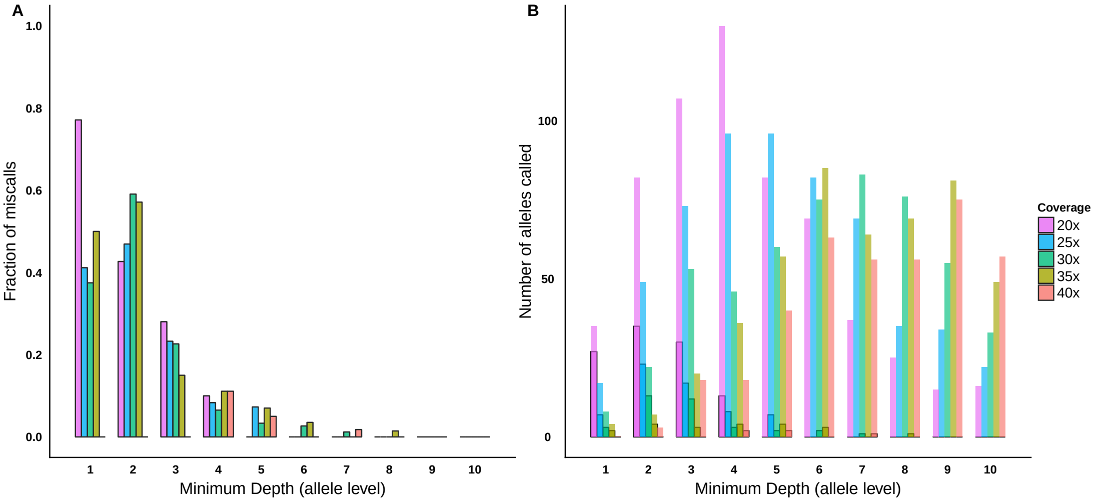

Figure S4. Incorrect calls at allele-specific minimum depths across varying coverages for 11 samples from 1000 Genomes. Panel A shows the fraction of incorrect calls at varying allele-specific minimum depths across varying coverages. Panel B shows the total number of incorrect calls (black border) in front of the total number of alleles (no border, lighter shade) called at varying allele-specific minimum depths across varying coverages. Minimum depths on the x-axis are only shown up to 10 as the calls with larger minimum depths are all correct.
